# Effects of Electroacupuncture with Dominant Frequency at SP 6 and ST 36 Based on Meridian Theory on Pain-Depression Dyad in Rats

**DOI:** 10.1155/2015/732845

**Published:** 2015-03-04

**Authors:** Yuan-yuan Wu, Yong-liang Jiang, Xiao-fen He, Xiao-yun Zhao, Xiao-mei Shao, Jun-ying Du, Jian-qiao Fang

**Affiliations:** Department of Neurobiology and Acupuncture Research, The Third Clinical Medical College, Zhejiang Chinese Medical University, Hangzhou 310053, China

## Abstract

Epidemic investigations reveal an intimate interrelationship between pain and depression. The effect of electroacupuncture (EA) on pain or depression has been demonstrated individually, but its effect on pain-depression dyad is unknown. Our study aimed to screen a dominant EA frequency on pain-depression dyad and determine the validity of acupoint selection based on meridian theory. The pain-depression dyad rat model was induced by reserpine and treated using EA with different frequencies at identical acupoints to extract a dominant frequency and then administrated dominant-frequency EA at different acupoints in the above models. Paw withdrawal latency (PWL), emotional behavior of elevated zero maze (EZM) test, and open field (OF) test were conducted. We found that 100 Hz EA at Zusanli (ST 36) and Sanyinjiao (SP 6) (classical acupoints for spleen-deficiency syndrome) were the most effective in improving PWL, travelling distance in the EZM, and maximum velocity in OF compared to EA with other frequencies; ST 36 and SP 6 were proved more effective than other acupoints beyond the meridian theory and nonacupoints under the same administration of EA. Therefore, we concluded that 100 Hz is the dominant frequency for treating the pain-depression dyad with EA, and acupoints on spleen and stomach meridians are preferable choices.

## 1. Introduction

Pain and emotional disorders are regarded as multidimensional structures which have received extensive attentions [1]. Suffering pain is a high risk factor for persistent depression [2–4], and approximately 35% of pain sufferers have comorbid depression [5]. On the other hand, depression is often accompanied with an increasing incidence of pain complaints. A clinical study indicates that 43.4% of patients with depression suffer from at least one painful physical symptom [6]. This complex relationship between chronic pain and depression is termed either a depression-pain syndrome or a pain-depression dyad [7–10]. Acupuncture, recognized worldwide for its analgesic effects, is extensively utilized for clinical patients with chronic pain [11]. Electroacupuncture (EA) performed at different frequencies exhibits different analgesic effects. Numerous studies have also established the validity of acupuncture on depressive emotions [12–14]. However, the effect of acupuncture on pain-depression dyad still remains unclear.

An animal model of the pain-depression dyad should include quantifiable physical pain and depressive symptoms. In order to explore its biochemical mechanisms or effective therapies, previous studies have used multiple depression models or pain models to mimic the pain-depression dyad [15–17]. Reserpine, as a commonly utilized pharmacological tool drug to establish a model of depression in rodents by irreversibly blocking the vesicular monoamine transporter and depleting the brain of the monoamines serotonin, norepinephrine, and dopamine [18], could produce a significant decrease in the overall nociceptive threshold and behavioral depression [19, 20]. This indicates that reserpine treatment may be used as an ideal model to study the pain-depression dyad. According to Traditional Chinese Medicine (TCM) symptomatology, reserpine treatment induces poor appetite, fatigue, depression, or irritability. These symptoms are core characteristics of spleen-deficiency syndrome, which may be treated by tonifying the spleen.

In this study, we hypothesized that the therapeutic effect of EA on the pain–depression dyad is dependent on the stimulation frequency or acupoint specificity. We tested the effects of EA at various frequencies (2 Hz, 15 Hz, 100 Hz, and 2/100 Hz) at acupoints Zusanli (ST 36) and Sanyinjiao (SP 6) (the two points for spleen-deficiency [21]) on the pain-depression dyad induced by reserpine. Secondly, we compared the effects of dominant frequency EA at ST 36 and SP 6 with other acupoints that are not in stomach and spleen meridians, as well as nonacupoints.

## 2. Materials and Methods

### 2.1. Animals

Male Sprague-Dawley rats (250 ± 20 g) were purchased from the SLAC (Shanghai Laboratory Animal Center, Shanghai, China) and housed in 40 cm × 50 cm × 25 cm cages with ad libitum access to food and water at room temperature (25 ± 1°C). The animals were housed in groups of 5-6 rats with a 12:12 light-dark cycle (dark cycle 8:00 PM–8:00 AM). All animal experiments were performed in accordance with the regulations of the State Science and Technology Commission for the Care and Use of Laboratory Animals (State Science and Technology Commission number 2, 1988).

### 2.2. Drugs

Reserpine was purchased from Sigma-Aldrich Co. LLC (Sigma, USA). 100 mg of reserpine powder was dissolved in 400 *μ*L glacial acetic acid and then diluted with distilled water to 20 mL. Animals in the experimental groups were subcutaneously injected with reserpine solution (0.2 mL/kg daily) for three consecutive days. Rats in the normal group were subcutaneously injected with vehicle solution (5% solution of glacial acetic acid in distilled water) (0.2 mL/kg daily) for three consecutive days.

### 2.3. Experimental Design

Two experiments were conducted. 

**Figure 1:**
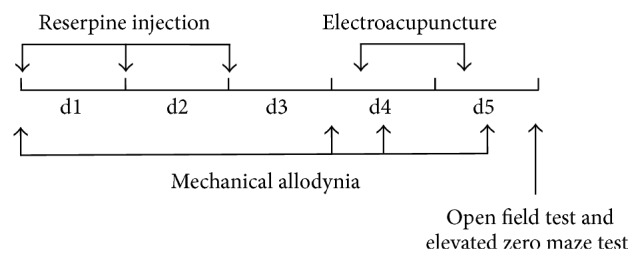
Experimental procedure. Right after the baseline pain threshold was tested, animals in the experimental groups and the normal group were administered reserpine injection or vehicle (0.2 mL/kg, subcutaneously), respectively, for three consecutive days. On the fourth day, PWL tests to examine mechanical allodynia were performed before and after the EA treatment, respectively. On the fifth day, another EA treatment was performed, followed by PWL test, EZM test, and OF test.

*Experiment 1*. EA frequency screening: in this experiment, all animals were randomly assigned to the normal group and the reserpine treatment group whose subgroups included model group and EA treatment groups with EA stimulation frequencies of either 2 Hz, 15 Hz, 100 Hz, or 2/100 Hz group (*n* = 10 per group). After assessing baseline pain threshold via paw withdrawal latency (PWL) to a mechanical stimulus, the reserpine solution was injected once daily for three consecutive days. PWL was assessed again 24 hours after the last reserpine injection. On the same day, EA treatment was performed and PWL was assessed 30 min later. On the fifth day, PWL, EZM test, and OF test were performed in sequence 30 min after EA treatment (Figure 1). 

*Experiment 2*. Effect comparisons of acupoint selections guided or not by meridian theory: all subjects were randomly divided into the normal group and the reserpine treatment group whose subgroups included model group, ST 36 + SP 6 group, LI 11 + TE 5 group, and nonacupoint group (*n* = 8 per group). The experimental procedure was performed as in Experiment 1 (Figure 1).

### 2.4. Electroacupuncture Method

In Experiment 1, bilateral Zusanli (ST 36, 5 mm lateral to the anterior tubercule of the tibia) and Sanyinjiao (SP 6, 10 mm proximal to the prominence of the medial malleolus) acupoints were selected. Stainless steel acupuncture needles (0.25 mm in diameter, 13 mm in length) were inserted into the acupoints at a depth of 5 mm. The two ipsilateral needles were connected with the output terminals of the HANS Acupuncture Point Nerve Stimulator (LH-202H, Huawei Co. Ltd., Beijing, China). The EA parameters were set as follows: square wave current output (pulse width: 0.2 ms); stimulation intensities of 1.0 mA, 1.5 mA, and 2.0 mA were used, each one for 15 minutes in sequence, for a total of 45 minutes. The stimulation frequencies were 2 Hz, 15 Hz, 100 Hz, and 2/100 Hz, as determined by treatment groups assignment. In the 2/100 Hz group, 3 sec 2 Hz and 3 sec 100 Hz stimulations were shifted automatically for 45 minutes [22]. All rats were loosely immobilized by a cloth cover with no physical restraint. EA was performed after the last reserpine injection on the fourth day and was repeated on the fifth day.

In Experiment 2, the ST 36 + SP 6 group received bilateral needles at Zusanli (ST 36) and Sanyinjiao (SP 6). The LI 11 + TE 5 group received bilateral needles at Quchi (LI 11, depression lateral to anterior elbow joints, on the proximal side of the radius) and Waiguan (TE 5, 3 mm above the wrist, between the ulna and radius). The nonacupoint group received needles at two bilateral points on the waist (not belonging to any acupoints and meridians). The dominant EA frequency was selected based on results from Experiment 1. EA method was identical to the previous experiment.

### 2.5. Assessment of Static Mechanical Sensitivity

The static mechanical allodynia was measured using the Von Frey test [23, 24]. An Electronic von Frey instrument (EVF-3; Bioseb, France) was employed in the von Frey test. Measurements ranged from 0 to 500 grams with 0.2 gram accuracy. Rats were placed on a metal mesh and allowed to adapt for 15 minutes. The stimulus was applied to the left hind paw for five seconds. The paw withdrawal latency (PWL) was defined as the minimum about of pressure needed to elicit a robust and immediate withdrawal reflex of the left hind paw and was measured before and after reserpine/vehicle injections and 30 minutes after each EA treatment. The pain threshold for each rat was the average of the three measured values.

### 2.6. Behavioral Test for Depressive-Like Behavior

Depressive-like behavior was quantified using the elevated zero maze (EZM) test and open field (OF) test, which are usually used to evaluate emotional state and activity of freedom movement [25, 26]. All exterior lights were blocked. The ambient noise in the testing room was maintained below 40 db, and abrupt loud noises that could alter locomotion or produce prolonged immobility were avoided during the test. The room's temperature was around 25°C.

#### 2.6.1. Elevated Zero Maze (EZM) Test

The maze with a black metallic annular platform (100 cm in diameter, 25 cm in width, and 55 cm in height) was divided equally into four quadrants. Two opposite quadrants (close arms) were enclosed by black metallic walls (30 cm high) on both the inner and the outer edges of the platform, while the remaining two opposite quadrants (open arms) were surrounded by nothing [26]. All subjects were placed in the testing room one hour before the test. The entire apparatus was wiped with 75% ethanol before each trial. The animal was placed in the center of a closed arm for 20 seconds to adapt the environment and then behavior was videotaped and quantified by the SMART 3.0 system for 5 minutes.

#### 2.6.2. Open Field (OF) Test

Four square black plexiglass open field arenas (100 cm × 100 cm × 50 cm) were placed together to form the apparatus, such that each side was 200 cm and the area of four arenas was included. The entire apparatus was wiped with 75% ethanol before each trial. All subjects were placed in the testing room one hour before the test to adapt the environment. At the beginning of the trial, each animal was firstly placed in the center of the arena for 20 seconds to adapt the environment, and then the next five minutes of behavior was videotaped and quantified by the SMART 3.0 system.

### 2.7. Statistical Analysis

All data were expressed as mean ± SEM. For significance evaluation, data sets with normal distribution were analyzed by one-way analysis of variance (ANOVA) followed by the least significant difference post hoc (LSD). *P* values < 0.05 were considered statistically significant.

## 3. Results

### 3.1. Effects of EA with Different Frequencies on Paw Withdrawal Latency in Rats with the Pain-Depression Dyad

**Figure 2:**
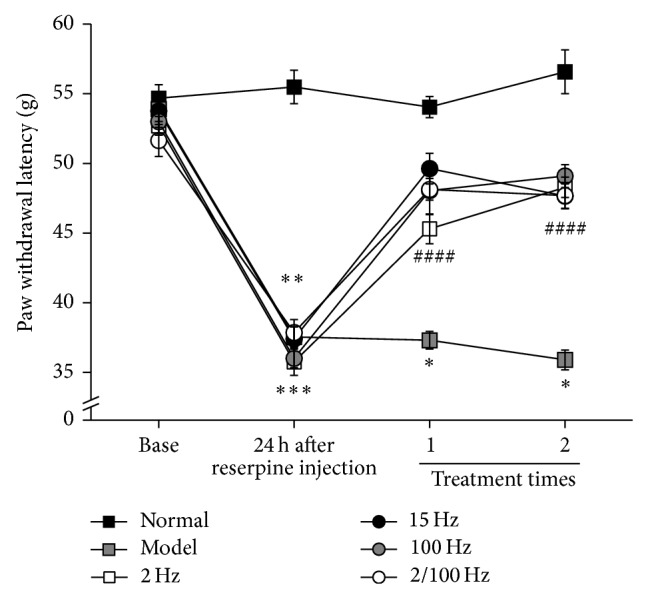
Effect of EA with different frequencies on paw withdrawal latency in rats with pain-depression dyad. The PWL was measured with an electronic von Frey apparatus (Bioseb, EVF3.0). Graphs represent the mean ± SEM (*n* = 10) in each group. ^*^ *P* < 0.05, compared to the normal group. ^#^ *P* < 0.05, compared to the model group.

To determine an effective EA frequency on the pain-depression dyad, stimulation frequencies of 2 Hz, 15 Hz, 100 Hz, and 2/100 Hz were employed in the experiment. As shown in Figure 2, compared to the normal group, reserpine treatment produced a significant decrease in PWL (*P* < 0.05), and significant increases in PWL were observed after EA with 2 Hz, 15 Hz, 100 Hz, and 2/100 Hz. However, there were no significant differences between EA groups at different frequencies (*P* > 0.05).

### 3.2. 100 Hz EA Improved the Depressive-Like Behavior in the Elevated Zero Maze Test

**Figure 3:**
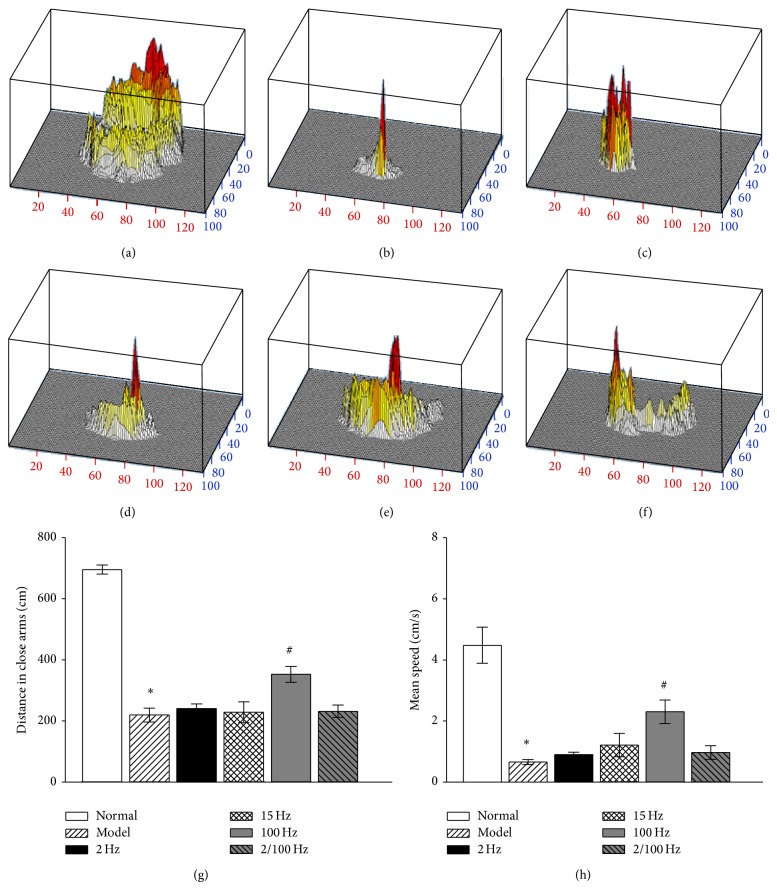
Effect of EA on the pain-depression dyad in elevated zero maze test. Columns represent the mean ± SEM of all 10 animals in each group. ^*^ *P* < 0.05, compared to normal group. ^#^ *P* < 0.05, compared to model group. (a)–(f) Three-dimensional activity of one rat in the open field from the normal group, model group, 2 Hz group, 15 Hz group, 100 Hz group, and 2/100 Hz group, respectively; (g) travel distance in the close arms in each group; (h) the mean speed in the entire maze for each group.

As shown in Figures 3(g) and 3(h), reserpine treatment significantly decreased distance travelled in the close arms and mean speed (*P* < 0.05), compared to the normal group. EA treatment with 100 Hz significantly increased distance travelled in the close arms and mean speed, compared to the model group (*P* < 0.05). However, this effect was not seen following EA treatment with 2 Hz, 15 Hz, or 2/100 Hz (*P* > 0.05).

### 3.3. 100 Hz EA Improved the Depressive-Like Behavior in Open Field Test

**Figure 4:**
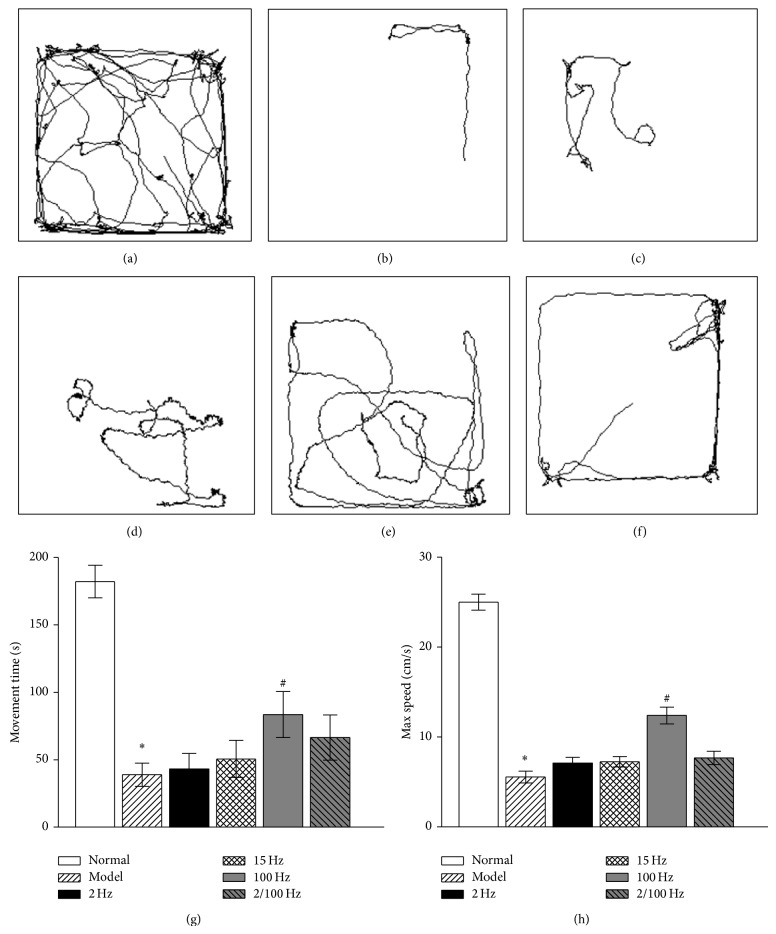
Effects of EA at different frequencies on the pain-depression dyad in open field test. ^*^ *P* < 0.05, compared to normal group. ^#^ *P* < 0.05, compared to model group. (a)–(f) Representative trajectories in the open field of the normal group, model group, 2 Hz group, 15 Hz group, 100 Hz group, and 2/100 Hz group, respectively; (g) time mobile in each group; (h) the maximum locomotor speed in the normal group and five experimental groups.

To further validate the antidepressant effects of EA treatment, we then performed the OF test to observe the effects of EA treatment on the pain-depression dyad in rats. As shown in Figures 4(a), 4(b), 4(g), and 4(h), compared to normal group, reserpine treatment significantly reduced distance travelled in the closed arms and maximum speed (*P* < 0.05). Figures 4(b)–4(h) display that EA treatment with 100 Hz significantly increased both time mobile and maximum speed compared to the normal group (*P* < 0.05), but EA treatment with 2 Hz, 15 Hz, and 2/100 Hz did not cause a significant change in either locomotion or maximum speed (*P* > 0.05).

### 3.4. Effects of EA at Different Acupoints Selections Guided or Not by Meridian Theory

As shown in Figures 3 and 4, 100 Hz EA treatment was most effective in producing both analgesia and antidepressant effects in the pain-depression dyad rat model. For this reason, the 100 Hz was employed as the exclusive EA frequency for Experiment 2.

**Figure 5:**
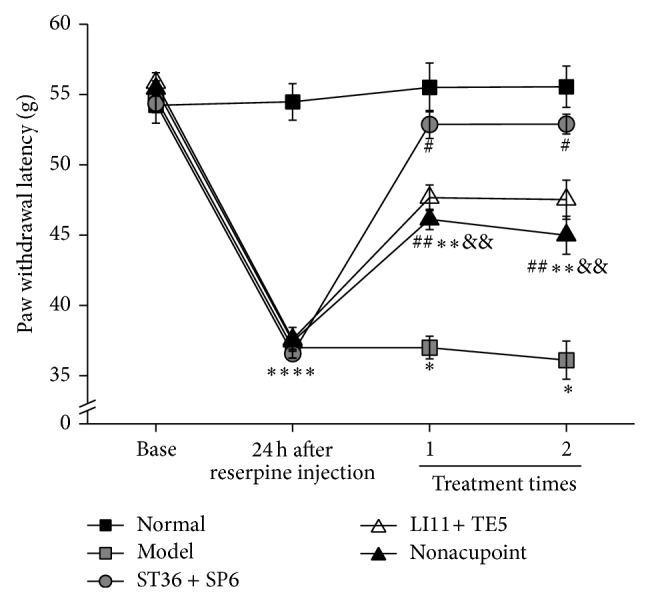
Effects of EA at different locations (100 Hz) on paw withdrawal latency in the pain-depression dyad model. The PWL was measured with an electronic von Frey apparatus (Bioseb, EVF3.0). Graphs represent the mean ± SEM of all 8 animals in each group. ^*^ *P* < 0.05 compared to normal group. ^#^ *P* < 0.05, compared to model group. ^&^ *P* < 0.05, compared to ST 36 + SP 6 group.

As shown in Figure 5, reserpine produced a significant decrease in PWL (*P* < 0.05) compared to the normal group. EA treatment at ST 36 + SP 6, LI 11 + TE 5, and nonacupoint locations significantly increases PWL, compared to the normal group (*P* < 0.05). EA treatment at ST 36 and SP 6 produced a significant increase in PWL compared to the LI 11 + TE 5 group and nonacupoint group (*P* < 0.05).

### 3.5. 100 Hz EA at ST 36 and SP 6 Attenuated Depressive-Like Behavior in the Elevated Zero Maze Test

**Figure 6:**
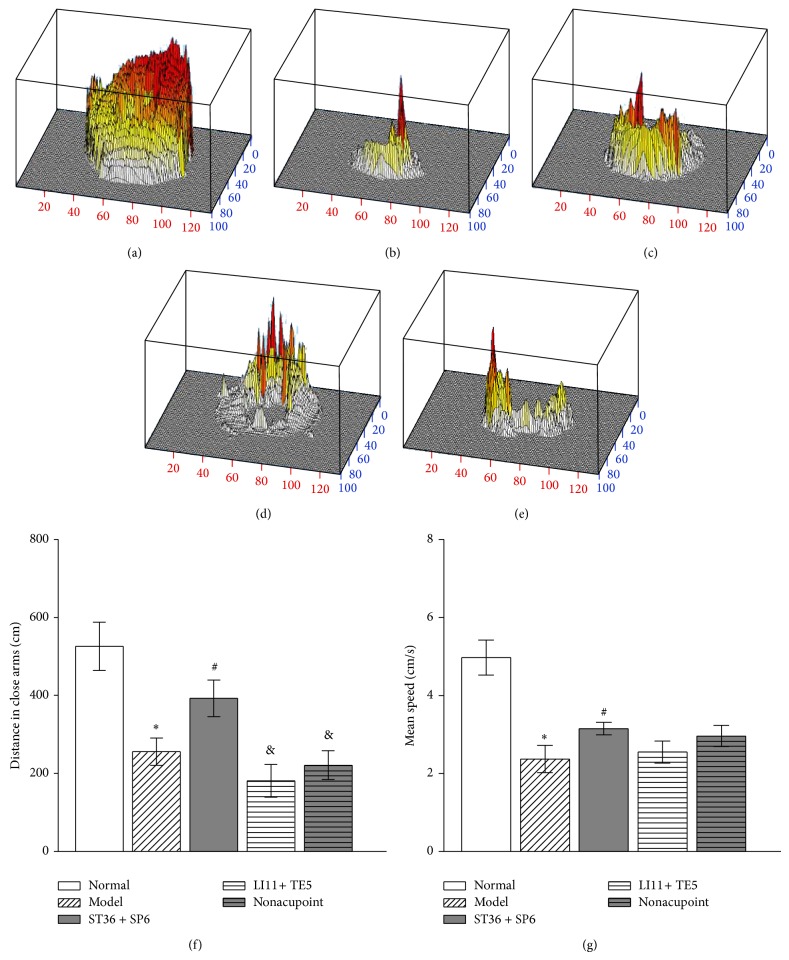
Effects of 100 Hz EA on elevated zero maze test in the pain-depression dyad model rats. Columns represent the mean ± SEM of all 8 rats in each group. ^*^ *P* < 0.05, compared to normal group (*P* < 0.05). ^#^ *P* < 0.05, compared to model group. ^&^ *P* < 0.05, compared to the ST 36 + SP 6 group. (a)–(e) Three-dimensional activity of one rat in the open field from the normal group, model group, ST 36 + SP 6 group, LI 11 + TE 5 group, and nonacupoint group, respectively; (f) distance travelled in the close arms; (g) the mean locomotor speed in the entire maze in each group.

As shown in Figures 6(a), 6(b), 6(f), and 6(g), compared to the normal group, reserpine treatment induced a significant decrease in distance travelled in the close arms and mean speed (*P* < 0.05). Figures 6(c)–6(g) showed that EA at acupoints ST 36 and SP 6 significantly increased distance travelled in the closed arms and mean speed compared to the model group. However, no significant increase was found in the LI 11 + TE 5 group and nonacupoint group (*P* > 0.05); the distance travelled in ST 36 + SP 6 group was significantly greater than that in LI 11 + TE 5 group and nonacupoint group (*P* < 0.05).

### 3.6. 100 Hz EA at ST 36 and SP 6 Attenuated the Depressive-Like Behavior in Open Field Test

**Figure 7:**
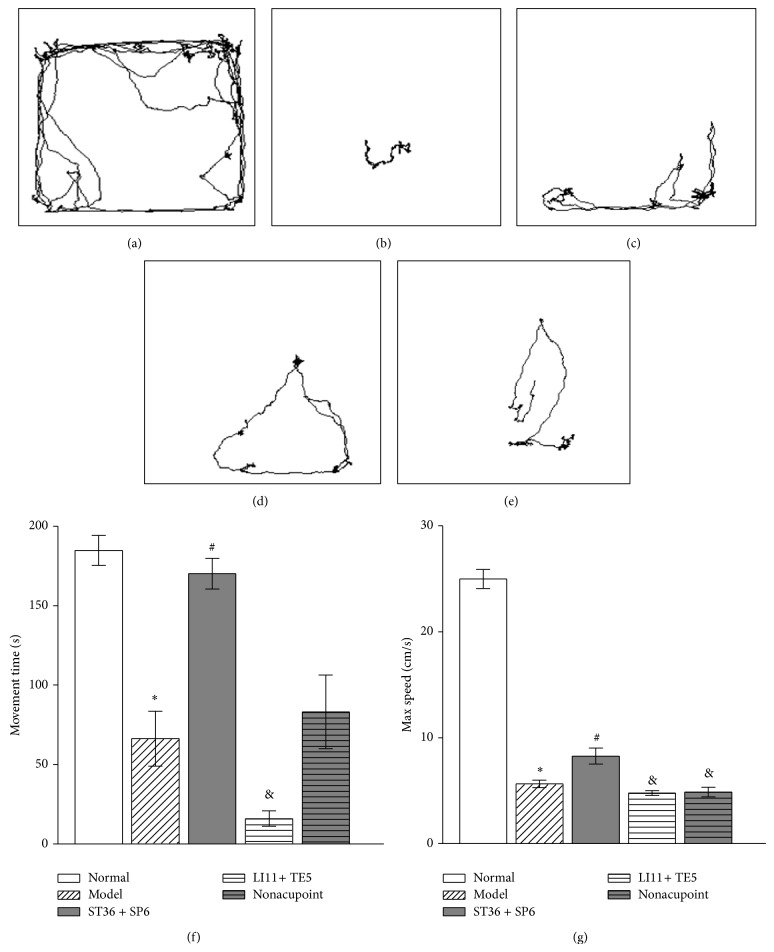
Effect of 100 Hz EA treatment on open field test in the pain-depression dyad model rats. Graphs represent the mean ± SEM of all 8 animals in each group. ^*^ *P* < 0.05, compared to normal group. ^#^ *P* < 0.05, compared to model group. ^&^ *P* < 0.05, compared to ST 36 + SP 6 group. (a)–(e) Examples of trajectory in the open field among the normal group, model group, ST 36 + SP 6 group, LI 11 + TE 5 group, and nonacupoint group, respectively; (f) average time mobile of each group; (g) the maximum locomotor speeds in each group.

Compared to the normal group, reserpine treated rats showed a significant decrease in movement time (Figures 7(a), 7(b), and 7(f)) and maximum speed (Figures 7(a), 7(b), and 7(g)) (*P* < 0.05). Compared to the model group, EA stimulations at ST 36 and SP 6 significantly increased the locomotion time and maximum speed (*P* < 0.05), but no significant changes were observed in LI 11 + TE 5 group and nonacupoint group (*P* > 0.05) (Figures 7(b)–7(g)). Furthermore, a significant increase in time mobile was observed in the ST 36 + SP 6 group compared to the LI 11 + TE 5 group (*P* < 0.05), and a significant increases in maximum speed was displayed in ST 36 + SP 6 group, compared to the LI 11 + TE 5 group and nonacupoint group (*P* < 0.05).

## 4. Discussion

Neurochemical evidence demonstrated that pain and depression appear to share common mechanisms such as dysfunction of monoaminergic systems. Reserpine-induced behavioral depression was caused by the depletion of brain monoamines and is usually ameliorated 72 hours after the drug is discontinued [20]. In the present study, rats which were injected with reserpine (0.2 mL/kg per day) subcutaneously for three consecutive days exhibited a diminished PWL in the electronic Von Frey test (mechanical allodynia), a measure of mechanical allodynia. Reserpine treatment also induced depressive-like behavior in EZM test as well as OF test. Taken together, this implies that a pain-depression dyad rat model was successfully produced by reserpine treatment. According to Traditional Chinese Medicine (TCM), the symptoms induced by reserpine treatment including poor appetite, fatigue, depression, or annoyance exhibited the characteristics of spleen-deficiency syndrome.

There have been many studies examining the frequency specificity of EA on pain modulation [27, 28], but it is not known whether the effect of EA on the pain-depression dyad is associated with stimulation frequency specifically. In the current study, EA stimulation frequencies of 2 Hz, 15 Hz, 100 Hz, and 2/100 Hz were applied at both bilateral ST 36 and SP 6 separately in a rodent model of the pain-depression dyad. We found that EA at all four frequencies exerted analgesia to different extents, but only 100 Hz EA treatment significantly recovered the depressive-like behavior. Although previous studies have demonstrated that EA treatment with 2 Hz is more effective than 100 Hz for the regulation of supraspinal opioid function in analgesia [29–31], we found that EA with 100 Hz most significantly improved negative behavioral symptoms in the reserpine-induced pain-depression dyad in which 5-HT in supraspinal central nervous system (CNS) plays an important role. The precise mechanism responsible for this phenotype will be further investigated in future studies.

Acupoints, one of most important factors in the therapeutic effects of EA, have been attracting more and more researchers' attention [32]. In the present study, reserpine treatment in rats induced symptoms resembling that of spleen-deficiency syndrome: poor appetite, fatigue, and depression. According to Traditional Chinese Medicine, spleen-deficiency syndrome should be treated by tonifying spleen; acupoints of the Spleen Meridian of Foot Taiyin and Stomach Meridian of Foot Yangming should be selected to reinforce the spleen [21]. SP 6 in Spleen Meridian and ST 36 in Stomach Meridian are usually targeted in acupuncture therapy to improve spleen-deficiency syndrome, according to the meridian theory [33]. LI 11 and TE 5 which are not in Spleen or Stomach Meridian but associated with analgesia, and points which are not from any meridian, were selected to be controls.

100 Hz EA treatment at points not from any meridian or acupoints was found to be effective in pain relief, suggesting nonspecific effects of EA, consistent with previous studies [34]. However, we found that 100 Hz EA treatment at ST 36 and SP 6 exhibited the most robust analgesic effect in the reserpine-induced pain-depression dyad model than treatment at LI 11 and TE 5 and points not from any meridian. Furthermore, 100 Hz EA at ST 36 and SP 6 was also the most effective in rescuing reserpine-induced depressive-like behaviors. These results verified the great significance of acupoint differentiation based on meridian theory in EA treatment of the pain-depression dyad. In the future studies, we will investigate specific mechanisms underlying why acupoint stimulation from the Spleen Meridian and Stomach Meridian is preferable for improving symptoms found in the pain-depression dyad model induced by reserpine.

## 5. Conclusion

In the reserpine-induced pain-depression dyad rat model, EA treatment with 100 Hz exerted the most significant effects on producing analgesia and attenuating depressive-like behavior. Electroacupuncture at ST 36 and SP 6, selected based on meridian theory, play a specific role in the improvement of both physical pain and depression.
